# Mental health symptom changes in pregnant individuals across the COVID-19 pandemic: a prospective longitudinal study

**DOI:** 10.1186/s12884-022-05144-6

**Published:** 2022-12-03

**Authors:** Lauren A. Gimbel, Amanda A. Allshouse, Dylan Neff, Robert M. Silver, Elisabeth Conradt, Sheila E. Crowell

**Affiliations:** 1 Department of Obstetrics & Gynecology, University of Utah Health, Salt Lake City, USA; 2 Department of Psychology, University of Utah, Salt Lake City, USA; 3 Departments of Psychiatry and Pediatrics, Duke University, Durham, USA; 4 Department of Psychiatry, University of Utah, Salt Lake City, USA

**Keywords:** (10 maximum): pregnancy, Postpartum, Perinatal mental health, Maternal mental health, Coronavirus, COVID-19, Pandemic, Mental health symptoms, Depression, Emotion dysregulation

## Abstract

**Background:**

Initial studies found that mental health symptoms increased in pregnant and postpartum individuals during the COVID-19 pandemic. Less research has focused on if these putative increases persist over time and what factors influence these changes. We examined the longitudinal change in mental health symptoms in pregnant and postpartum individuals and investigated moderation by maternal emotion dysregulation and the incidence of coronavirus.

**Methods:**

Pregnant and postpartum individuals at the University of Utah were invited to join the COVID-19 and Perinatal Experiences (COPE) Study. Beginning on April 23, 2020 participants were sent a survey comprised of demographics, medical and social history, pregnancy information and self-assessments (Time 1). Participants were contacted 90 days later and invited to participate in a follow-up questionnaire (Time 2). Daily coronavirus case counts were accessed from the state of Utah and a 7-day moving average calculated. Within-subject change in mental health symptom scores, as measured by the Brief Symptom Inventory, was calculated. Linear mixed effects regression modeling adjusted for history of substance abuse and mental health disorders.

**Results:**

270 individuals responded between April 23rd, 2020 and July 15th, 2021. Mental health symptom scores improved by 1.36 points (0.7-2.0 p < 0.001). The decrease in mental health symptoms was not moderated by the prevalence of COVID-19 cases (p = 0.19) but was moderated by emotion dysregulation (p = 0.001) as defined by the Difficulties in Emotion Regulation Scale short form. Participants with higher emotion dysregulation also had higher mental health symptom scores.

**Conclusion:**

Mental health symptoms improved over the course of the pandemic in the same pregnant or postpartum participant. Our findings do not negate the importance of mental health care during the pandemic. Rather, we believe this identifies some aspect of resiliency and adaptability. Examining emotion dysregulation, or asking about a history of mental health, may be helpful in identifying persons at higher risk of heightened responses to stressors.

## Introduction

The COVID-19 pandemic has been detrimental to both physical and mental health. Initial studies found an increase in anxiety and depression symptoms in pregnant individuals during the COVID-19 pandemic [1–10]. However, these studies did not examine prospective symptoms over time during the pandemic and we do not know if the initial stress response and heightened symptoms persist as the pandemic continues or what factors influence these changes.

Modeling has suggested that COVID-19 fear and anxiety from the pandemic may be related to mental health symptoms [11]. As time goes on from the beginning of the pandemic and the initial acuity of the stress becomes chronic, it is possible that mental health symptoms could improve, more so in some individuals than others. However, changes in daily life, quarantine and isolation are thought to influence mental health symptoms [12]. In the state of Utah a tiered response to the pandemic was created which was based on case counts. As case counts increased, government mandates also increased. Additionally, rising case counts of a transmittable disease may contribute to the acuity of the pandemic and moderate changes in mental health symptoms.

Emotion dysregulation, how an individual experiences and expresses emotion, [13]. may also affect the response to a stressful situation like a pandemic. Emotion dysregulation can be measured by self-report with the Difficulties in Emotion Regulation Scale (DERS) [14, 15]. As a transdiagnostic measure, the DERS is a stronger predictor of emotional distress than single-diagnostic measures [16]. While information in pregnancy is limited, it is predictive of maternal sensitivity and challenging mother-infant interactions as early as six months postpartum [17]. Pregnant individuals with high emotion dysregulation may have greater difficulty responding to the stress of the pandemic, which may result in more mental health symptoms.

The purpose of this study was to determine how mental health symptoms in pregnant or postpartum individuals change over the time course of the pandemic, and if the change is moderated by the number of new COVID-19 cases or by maternal emotion dysregulation. We hypothesize that over the course of the pandemic mental health symptoms will improve, and both state prevalence of COVID-19 cases and emotion dysregulation will moderate the severity of mental health symptoms over time.

## Methods

### Study population

The COVID-19 and Perinatal Experiences (COPE) Study is a prospective cohort of pregnant and postpartum individuals surveyed during the COVID-19 pandemic. The COPE Study was initially developed at NYU Langone Health and internationally distributed as a part of the COVGEN Research Alliance, a partnership that supports global research on the COVID generation (those born and growing up during the COVID-19 pandemic). The goal of the COPE study was to examine stress and emotional experiences of pregnant and postpartum individuals during the COVID-19 pandemic. We obtained IRB approval and all methods were carried out in accordance with relevant guidelines and regulations. Electronic informed consent was required from each participant. In the present analysis, we included participants who enrolled at a single academic institution.

Pregnant and postpartum individuals were identified at the University of Utah health system through electronic medical records using International Classification of Disease (ICD) codes. Potential participants were eligible if they were over 18 years old, pregnant or less than 6 months postpartum and spoke English or Spanish. Eligible participants were contacted by email starting April 23, 2020. Posters and marketing materials were placed throughout clinics to improve recruitment. Individuals who did not have an email address were not contacted by an alternative method due to IRB regulations at our institution. Research Electronic Data Capture (REDCap) was used for survey administration, data collection and electronic consent. After initially filling out the survey (Time 1), participants were contacted via email 90 days later and invited to participate in a follow-up questionnaire (Time 2).

Participants who responded at both Time 1 and Time 2 before July 15th, 2021 were included in our analysis. Individuals were excluded if they had already responded to the COPE survey and were responding in an additional pregnancy or if they did not respond to enough items for a DERS score to be calculated. No more than one scale item could be missing to calculate a DERS score.

### Measures

A Mental Health Symptom Score (MHSS) was created using 9 items from the 18-item Brief Symptom Inventory (BSI-18). The BSI-18 examines depression, anxiety and somatization with validity tested in multiple types of patient populations including primary care [18–23]. The number of items from the BSI-18 was decreased to reduce participant burden with items being chosen to represent a range of symptoms (Table 1). Participants were asked how often they were distressed by the item in the past 7 days. Items were scored 1–5 (not at all – extremely), an average score was calculated, and the score was multiplied by 9 to create a total score ranging from 9 to 45.

**Table 1:** Mental Health Symptom Score (MHSS): for each of the following items participants were asked "in the past 7 days including today, how often were you distressed by..." with items scored 1-5 (not at all-extremely)

Item	Description
1	Feeling no interest in things
2	Nervousness or shakiness inside
3	Feeling lonely
4	Feeling tense or keyed up
5	Nausea or upset stomach
6	Feeling blue
7	Suddenly scared for no reason
8	Feeling hopeless about the future
9	Feeling fearful

Emotion dysregulation was defined using the Difficulties in Emotion Regulation Scale Short Form (DERS-SF) [15]. The DERS has been used in pregnancy with good internal consistency [24]. A higher score denotes higher emotion dysregulation. Coronavirus case counts per day were accessed from Utah Department of Health and a 7-day moving average calculated and appended to participant data by date of survey completion [25].

A history of mental health disorders was defined as a self-report of any of the following: history of mood and/or anxiety disorder, currently or previously receiving treatment for mental health concerns, or selecting the answer choice “I have had mental health concerns but have not been treated”. A history of substance abuse was defined as a self-report of untreated substance abuse concerns at any time, or previously receiving treatment for substance abuse.

Self-reported data were obtained through the COPE survey [26]. These data included demographic information, pregnancy details, medical history, social history, experiences related to COVID-19, and self-assessments. In late November 2020, the questionnaire used at Time 2 was modified to a condensed version to reduce participant burden.

### Statistical analysis

To determine how mental health symptoms in pregnant or postpartum individuals changed over the time course of the pandemic, a difference in MHSS was tested with a paired t-test. The MHSS was then modeled as a function of calendar time and participant response (Time 1 and Time 2). This model accounted for two observations from each individual with different responses to each variable (i.e. two responses for MHSS, two responses for DERS-SF) in a linear mixed effects regression model.

To determine whether change in mental health symptoms in pregnant or postpartum individuals was moderated by the number of new COVID-19 cases, we added an interaction between COVID-19 prevalence and time to the previously described model. To determine whether change in mental health symptoms was moderated by emotion dysregulation, we added an interaction between emotion dysregulation and time to the previously described model. History of mental health disorders and history of substance abuse, both identified a-priori, were considered for further effect modification in a sensitivity analysis.

This data analysis was generated using SAS software, Version 9.4 of the SAS System for Windows, SAS Institute Inc. Cary, NC. Study data were collected and managed using REDCap electronic data capture tools hosted at the University of Utah [27]. Graphics were created using GraphPad Prism version 9.1.2 for Windows, GraphPad Software, La Jolla California.

## Results

**Fig. 1:**
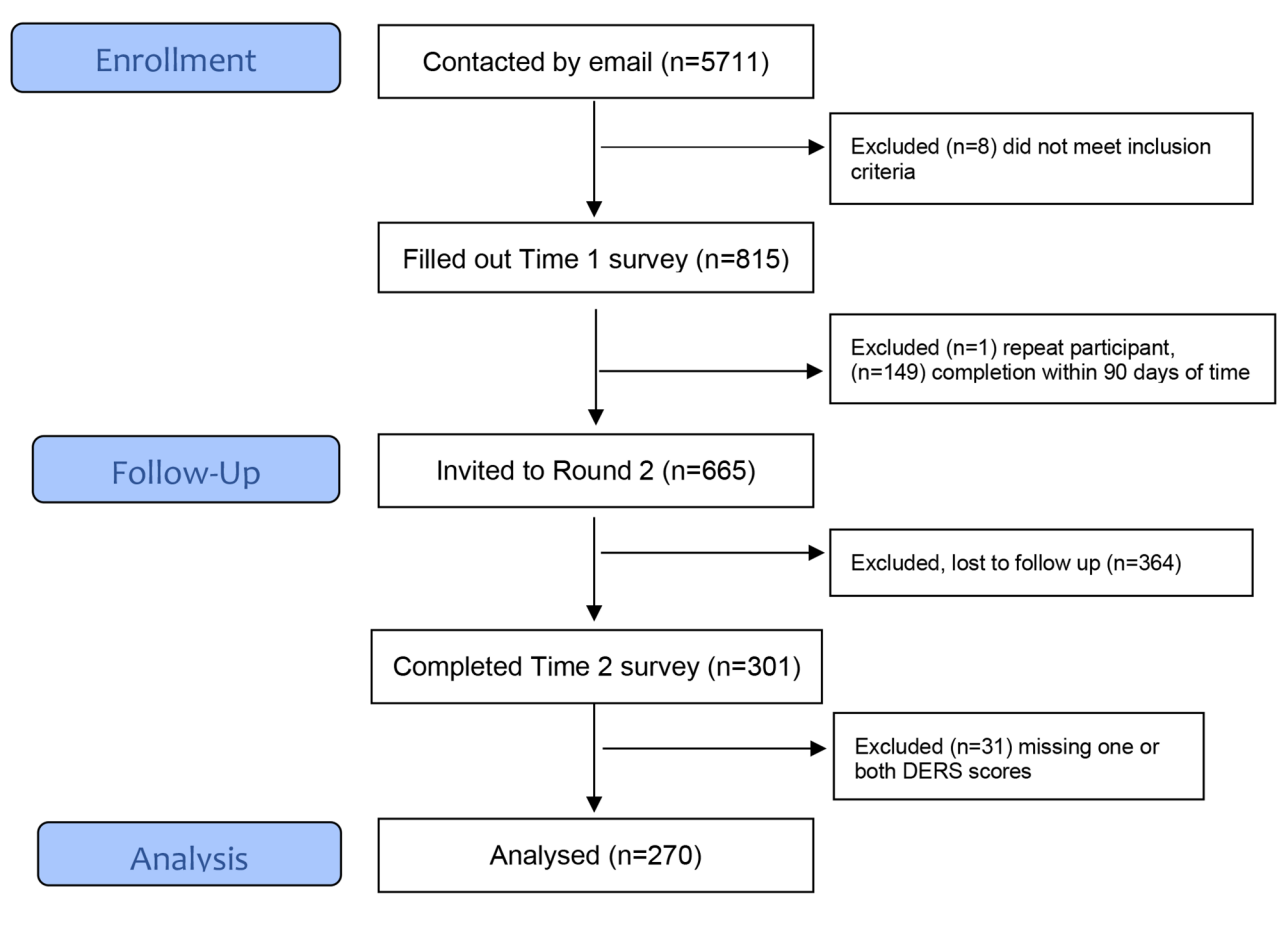
Flowchart

There were 270 individuals who met inclusion criteria (Fig. 1). Time 1 surveys were completed between April 23, 2020 and March 22, 2021. Time 2 surveys were completed between July 22, 2020 and July 15, 2021. Demographics and clinical characteristics of participants are included in Table 2. The cohort was mostly English speaking (99.6%) and white (82%). The average age was 31, the average BMI was 28, and the average gestational age at the time of first survey completion was 25 weeks. At Time 1 all participants were pregnant, at Time 2 most participants were postpartum (60%). A history of mental health disorders was reported by 57% of respondents, and a history of substance abuse was reported by 2%.

**Table 2:** Demographics and clinical characteristics

Item	N = 270 (%)
Language English Spanish	269 (99.6) 1 (0.4)
Race/Ethnicity Latin or Hispanic Black AI/AN/NHOPI Asian White Other or missing	27 (10.0) 1 (0.4) 4 (1.5) 12 (4.4) 220 (81.5) 6 (2.2)
Insurance^c^ Public (Medicare or Medicaid) Private None Unavailable	13 (4.8) 255 (94.4) 3 (1.1) N=1
Housing Studio 1 bedroom Multi-bedroom Unstable Decline to answer	2 (0.7) 19 (7.0) 245 (90.7) 2 (0.7) 2 (0.7)
Employment^c^ Working full-time Working part-time Looking for a job Student Unemployed Stay at home Retired Other	163 (60.4) 50 (18.5) 10 (3.7) 21 (7.8) 18 (6.7) 45 (16.7) 1 (0.4) 5 (1.9)
**Visit 1 Item**	**N = 270 (%)**
Age 18–24 25–34 ≥35 Unavailable	31.4 (30.9, 31.9)* 12 (4.5) 184 (69.2) 70 (26.3) N = 4
Currently pregnant	270 (100.0)
Gestational Age <13 weeks 13–27 weeks ≥27 weeks	25.1 (24.0, 26.2)* 20 (7.4) 103 (38.1) 147 (54.4)
BMI Not obese PreObese Obesity Class I Obesity Class II Obesity Class III Unavailable	27.9 ( 27.3, 28.5)* 80 (30.0) 100 (37.5) 58 (21.7) 17 (6.4) 12 (4.5) N = 3
Relationship Status Single Partnered/Married Divorced Unavailable	4 (1.5) 264 (98.1) 1 (0.4) N = 1
Mental Health Disorder History^a^	153 (56.7)
Substance Abuse History^b^	5 (1.9)
**Visit 2 Item**	**N = 270 (%)**
Weeks between Time 1 and 2	13.3 (13.0, 14.4)♦
Currently pregnant	108 (40.0)
Gestational Age 13–27 weeks ≥27 weeks	31.5 ( 30.5, 32.4)* 20 (18.5) 88 (81.5)

Values presented as frequency (percentage) unless stated otherwise

Abbreviations: AI = American Indian, AN = Alaska Native, NHOPI = Native Hawaiian or Other Pacific Islander

*Geometric Mean (95%CI)

♦Median (P25, P75)

^a^Mental Health Disorder History was defined as self-report of any of the following four criteria: (1) History of mood and/or anxiety disorder(2) currently receiving treatment for mental health concerns (3) received treatment for mental health at any time in the past or (4) I have had mental health concerns but have not been treated

^b^Substance Abuse History was defined as self-report of untreated substance abuse concerns at any time, or receiving treatment for substance abuse (including problems with prescription drugs, illegal drugs or alcohol) at any time

^c^Categories overlapped

**Fig. 2:**
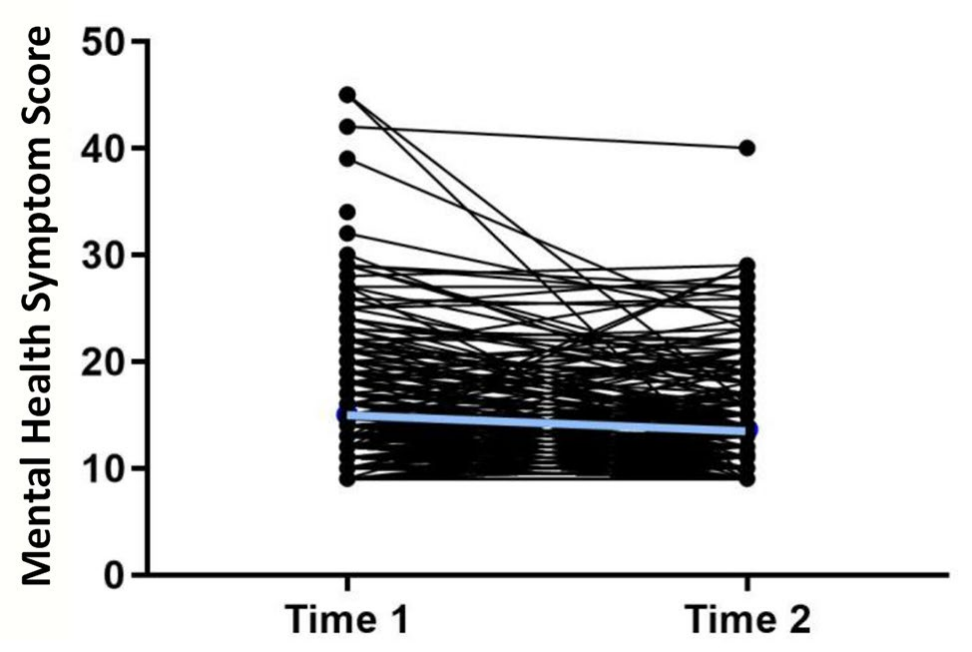
MHSS within Participants over time. Comparing paired MHSS responses, at time 2 MHSS was significantly lower on average as indicated by the blue line (1.36 decrease, (0.7-2.0 95% CI) p < 0.001)

**Fig. 3:**
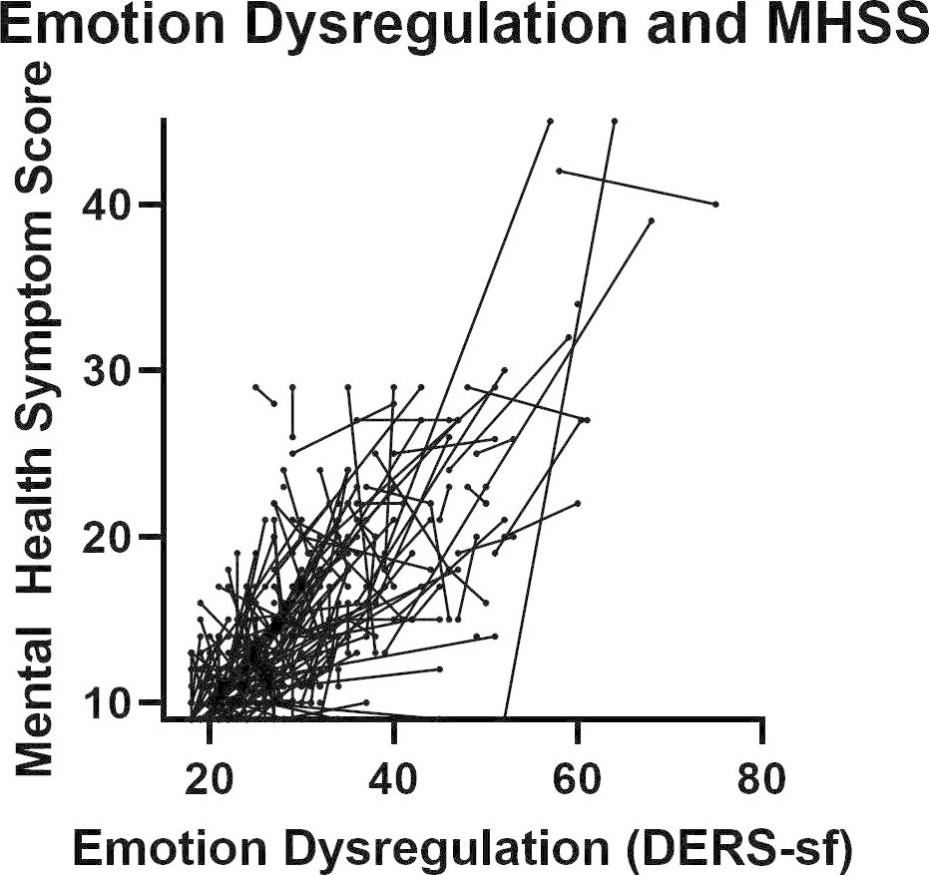
Emotion Dysregulation and MHSS Moderation. There is a positive association between MHSS and emotion dysregulation, with higher emotion dysregulation (higher DER-SF) there are higher MHSS

The prevalence of COVID-19 cases in the state of Utah were at their highest from November 2020-March 2021. Significant improvement in mental health symptoms were observed between the first and second response from participants in a comparison of paired observations (1.36 decrease, (0.7-2.0 95% CI) p < 0.001) (Fig. 2). In a linear mixed effects regression model, mental health symptoms decreased by 0.95 (0.12–1.78 95% CI) p = 0.02; this was not moderated by the prevalence of COVID-19 cases (p = 0.19) in the state of Utah but was moderated by emotion dysregulation (p = 0.001). Participants with higher emotion dysregulation also had higher mental health symptom scores (Fig. 3).

**Fig. 4:**
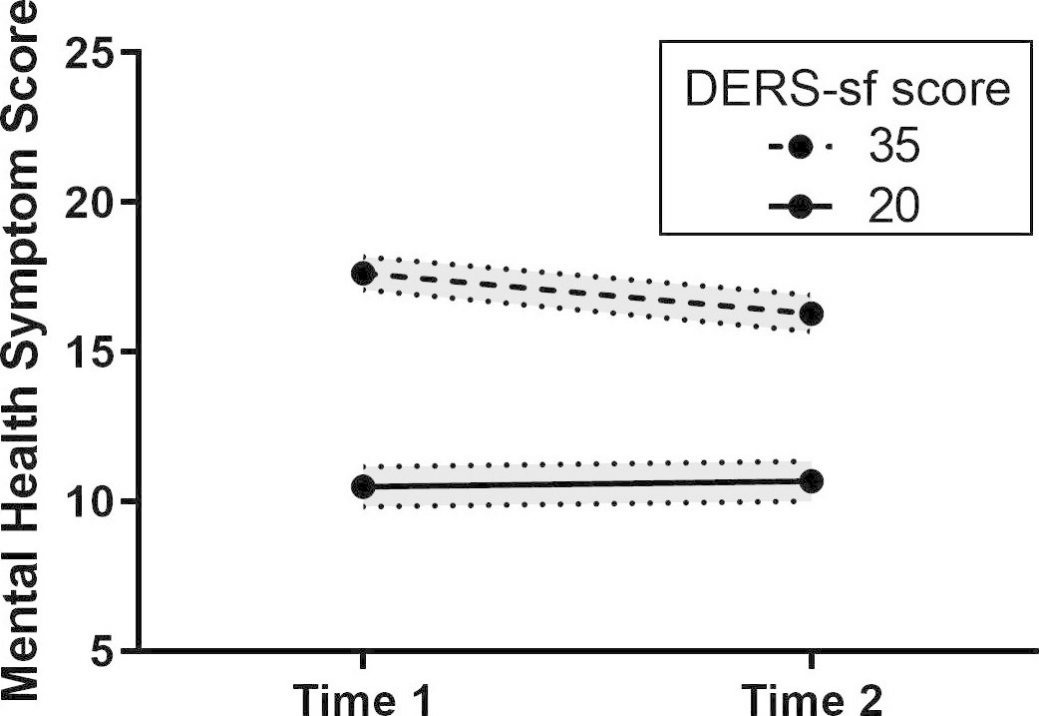
Emotion Dysregulation and MHSS Moderation Analysis by Time. Emotion dysregulation moderated the association between MHSS and time in the pandemic. DERS-SF 35 = 1 SD above the mean 75th percentile, DERS-SF 20 = 1 SD below the mean 25th percentile

From Time 1 to Time 2, a low DERS-SF score (low emotion dysregulation) was associated with a small change in mental health scores, while a high DERS-SF score (high emotion dysregulation) was associated with a greater decrease in mental health scores across survey times (Fig. 4). There was no significant difference in emotion dysregulation between participants who completed both Time 1 and Time 2 and participants who completed only Time 1.

**Fig. 5:**
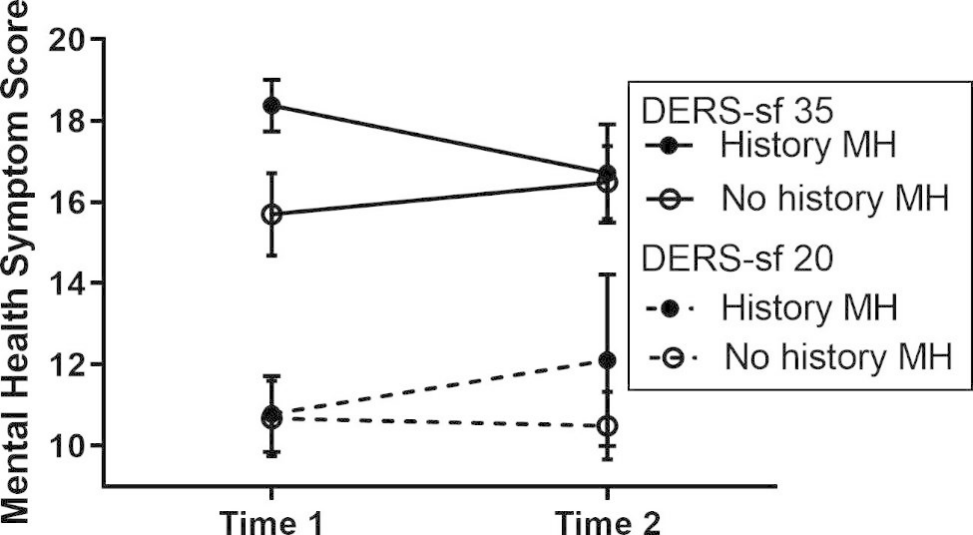
History of Mental Health Disorder (MH), Emotion Dysregulation and MHSS Moderation Analysis by Time. There was a significant difference in association with MHSS over time by DERS-SF and a history of mental health disorder. Significant improvement in MHSS in those with history of mental health disorder and high DERS-SF. Remainder were not significant

A sensitivity analysis was performed adding a self-reported history of mental health disorders to the model. History of a mental health disorder was a significant effect modifier for the association between emotion dysregulation, time, and mental health symptoms (p = 0.002). Participants with a history of mental health disorders and high emotion dysregulation had significant improvement in mental health symptoms over time, while those without a history of mental health and/or low emotion dysregulation did not have significant differences over time in mental health symptoms (Fig. 5).

## Discussion

Mental health symptoms in the same perinatal participant improved over the course of the pandemic. We did not find a moderating effect of Utah State COVID-19 cases on mental health symptoms over time. This could be due to an overestimation that people are influenced by the prevalence of cases in their state, people are affected more by their local community than the overall state, or that national or global news was more influential than local rates. Our findings of improvement in mental health symptom scores from Time 1 to Time 2 among individuals with higher emotion dysregulation may identify a group who are vulnerable to stressors but over time are able to self-regulate. It is important to note that mental health symptoms were higher among participants with higher emotion dysregulation regardless of survey time. In comparison, those with low emotion dysregulation started with low MHSS at Time 1 and retained low MHSS at Time 2. Their ability to regulate their emotions more effectively may help them maintain lower levels of distress.

The longitudinal nature of our data allowed us to examine the same participants over time during the pandemic. Prior studies differed in that they either examined different participants over time, participants from before to during the pandemic, or compared pregnant to non-pregnant individuals [28–31]. Since conception of our study a few others have been published. Daly and Robinson 2021 examined mental health symptoms from March to July 2020; after an initial increase in psychological distress, symptoms then improved [32]. These results are similar to our findings, over the course of the pandemic mental health symptoms improved in the same participant over time. A recent meta systematic review and meta-analysis by Robinson et al. compared longitudinal studies of mental health symptoms from before the pandemic to during the pandemic. When compared to pre-pandemic mental health symptoms, the initial increase in early pandemic mental health symptoms (March-April 2020) declined over time (May-July 2020) [33]. Our study began April 23rd 2020 and likely missed this initial increase that occurred acutely that was seen in other studies or that was identified by comparing pre-pandemic mental health symptoms to those during the initial months of the pandemic [28–31]. Instead our data likely only captured the decrease that was seen over a longer time period after the start of the pandemic similar to findings in the meta-analysis by Robinson et al [32, 33]. The longer nature of our study supports a continued pattern of likely sustained mental health improvements.

While only speculative, we believe our findings identify some aspect of resiliency and adaptability through the pandemic. It may relate to the difference in an acute compared to a chronic stressor, and supports the concept of habituation with chronic stressors. It may also point to the possibility of higher scores regressing toward the mean. Clinically, examining emotion dysregulation throughout the perinatal period may be helpful in identifying individuals earlier on who may be at higher risk of mental health symptoms or mood disorders triggered by psychosocial changes [34]. Additionally, the sensitivity analysis demonstrated that mental health symptom improvement over time was affected by a self-reported history of mental health disorders. This finding supports the clinical importance of obtaining a mental health history in risk stratifying patients, and emphasizes the importance of including a history of mental health disorders in research studies examining perinatal mental health.

This study has many strengths. It was a prospective cohort study prone to less bias than other observational studies. It also examines a large natural disaster in a longitudinal manner. A wide array of information was available to us, allowing us to control for confounding factors such as mental health history and substance abuse.

There are also limitations in our study. Measures of mental health symptoms are screening tools and cannot diagnose mental health disorders. We were not able to use the full BSI as the surveys in this study were condensed to reduce participant burden. While other studies have used condensed versions of the BSI, the 9 items that were included to create the MHSS is not a validated scale. These items were chosen to represent a range of symptom types and displayed high internal consistency (T1 α = 0.887, T2 α = 0.863). Utah state COVID-19 case counts served as a proxy for governmental restrictions but given differences in counties may not accurately represent an individual’s experience of fear and anxiety from the pandemic. Other limitations include those inherent in survey data with considerable selection bias, recall bias, response errors, and sample size limitations. While we recruited both pregnant and postpartum individuals, only currently pregnant individuals participated at Time 1 and most were postpartum at Time 2. Thus, findings may not be generalizable to the overall population.

## Conclusion

The pandemic has been detrimental in many regards, including psychologically for pregnant and postpartum individuals. However, mental health symptoms may improve over time. We recommend future studies on acute and chronic stressors, that examine whether and how habituation to the stressor could impact pregnancy outcomes. Screening tools, such as the DERS, or asking about a history of mental health, are helpful in identifying individuals at higher risk of heightened responses to stressors and in the future may allow work across disciplines to begin changing emotion regulation abilities to modify interactions with the future child.

## Data Availability

The datasets used during the study are available from authors on reasonable request.

## Abbreviations

COPE: COVID-19 and Perinatal Experiences Study
ICD: International Classification of Disease codes
REDCap: Research Electronic Data Capture
MHSS: Mental Health Symptom Score
BSI-18: Brief Symptom Inventory 18 item
DERS: Difficulties with Emotion Regulation Scale
DERS-SF: Difficulties with Emotion Regulation Scale Short Form
